# Quantitative Electroencephalography as a Complement to Symptom-Based Psychiatric Diagnosis: A Narrative Review

**DOI:** 10.3390/jpm16050261

**Published:** 2026-05-13

**Authors:** Francesco Amico, Scott Shannon, Steve Rondeau

**Affiliations:** 1 Neotherapy, Second Level, 2225 N Commerce Pkwy Suite #6, Weston, FL 33326, USA; 2 Texas Center for Lifestyle Medicine, 333 West Loop N. Ste 250, Houston, TX 77024, USA; 3 Department of Psychiatry, University of Colorado, Aurora, CO 80045, USA; scott@wholeness.com; 4 Wholeness Center, 2620 East Prospect Road, #190, Fort Collins, CO 80525, USA; 5 Axon EEG Solutions, Fort Collins, CO 80528, USA

**Keywords:** psychiatric assessment, quantitative electroencephalogram, qEEG, neurophysiological markers, adjunctive diagnosis psychiatry, qEEG diagnostic clarification

## Abstract

Background: Psychiatric assessments traditionally rely on the Diagnostic and Statistical Manual of Mental Disorders (DSM) for diagnostic guidance. This approach, however, is heavily based on the identification of cluster symptoms assessed through subjective interviews and questionnaires, without adequately controlling for overlapping symptoms or symptom specificity. This may lead to broad and often inaccurate diagnoses that overlook the patient’s unique experience and underlying neurobiological imbalances. As mental healthcare strives to move towards personalized medicine, incorporating more objective and precise measures of neuropsychological distress, it is essential to reduce the diagnostic and treatment inaccuracies that may stem from relying solely on empirical guidelines. This narrative review examines the limitations of the current approach and considers the potential role of quantitative electroencephalography (qEEG) as an adjunctive method that may enrich existing diagnostic processes. Methods: A structured literature search was conducted in Europe PMC on 31 January 2026. Original human studies and clinical trials in English with available abstracts were thematically selected. Results: The search yielded 1934 records, from which a focused subset of studies was selected based on direct relevance to the review themes. Conclusions: Integrating qEEG methods into traditional assessments could enhance diagnostic accuracy in psychiatric care and reduce patients’ exposure to inadequate treatments, ultimately leading to improved treatment outcomes and patient satisfaction.

## 1. Introduction

The Diagnostic and Statistical Manual (DSM) includes the most established guidelines for mental health assessment and is employed by all licensed mental health professionals around the world [1,2,3,4]. However, accumulating advances in translational and precision medicine suggest that interview-based evaluations of mental health pose considerable limitations, mainly because the approach heavily relies on identifying psychiatric disorders as syndromic entities rather than utilizing objective data. As a result, it often falls short in delivering accurate diagnoses or, importantly, guiding effective treatments [5,6]. Importantly, the DSM faces a challenge in its approach to categorizing mental health conditions based solely on symptoms without distinguishing the underlying neurobiological causes that may be shared among various psychiatric disorders [7,8,9]. Employing multimodal screening methods could be key to developing more precise and tailored treatments, saving patients from undergoing the deleterious trial-and-error process typically employed by mental health professionals.

Over the past few decades, there has been notable progress in the development of potential biomarkers in psychiatry, driven by advancements in neuroimaging, genomics, and molecular biology [10,11], although most approaches still require further validation prior to being integrated into routine clinical practice. These advances mark a significant departure from symptom-based assessments toward a more objective, quantifiable approach. In this context, techniques such as functional magnetic resonance imaging (fMRI) and electroencephalography (EEG) have shown promise in identifying the neural signatures associated with various psychiatric conditions [10]. However, standard brain imaging techniques can only identify brain anomalies at the population level, and their clinical application still awaits validation [12]. Moreover, the equipment and maintenance costs associated with brain imaging are generally not affordable to small clinical practices and clinics [13]. On the other hand, numerous studies spanning decades support the value of EEG methods in mental healthcare [14,15]. Recent research studies have demonstrated that modern EEG recording packages [16,17] exhibit promising accuracy, suggesting that, if combined with advanced data analysis tools, they might help compile a more comprehensive profile of brain activity by benchmarking the collected data against findings reported in the scientific literature [18,19]. More recently, it has been proposed that the convenience of EEG-based methods might be further enhanced by the introduction of dry, wireless EEG systems, making EEG data acquisition a practical option in various recording settings [20,21,22]. Importantly, the light weight of sensors and the flexibility in head movement restrictions of modern EEG equipment may contribute to achieving greater signal quality in younger persons [23], which could play an important role in detecting disorders at the prodromal stage. Finally, the low cost and portability of modern equipment make EEG-based assessments more affordable for the average psychiatric clinic, although signal detection/processing and artifact removal still await standardization across different montages and commercially available platforms [24,25].

While traditional EEG recordings are limited to analyzing waveform-only tracings in the 1–70 Hz range, modern EEG processing equipment can provide much more detailed information. A method referred to as quantitative EEG or qEEG [26,27] identifies functionally meaningful measures by reducing the high complexity and dimensionality of EEG data, typically recorded at hundreds to thousands of samples per second employing tens of scalp channels, to a few summary measures at most. Further, while standard EEG evaluations do not generate data on inter-regional interactions, which are key to cognition and consciousness [28], qEEG-based investigations can detect network dysfunctions and altered functional connectivity between distant cortical districts [29,30]. By employing intelligent mathematical algorithms, the qEEG method transforms raw EEG data into a representation of its frequency content, generating a continuous EEG “power spectrum”. The transformed data are then compared to referenced literature relative to age- and sex-matched healthy individuals [31]. 

The rhythmic EEG spectrum can be subdivided into five main oscillatory frequency bands: (1) delta (1–4 Hz) typically recorded during sleep, (2) theta (4–8 Hz) reflecting a state of drowsiness and also recorded during sleep, (3) alpha (8–12 Hz) occurring during periods of relaxation, (4) beta (12–30 Hz) commonly linked to the activation of cognitive processes, and (5) gamma (30–50 Hz or higher), driving both perception and synchronization across separate brain regions [32,33]. The qEEG algorithms calculate frequency-specific EEG power at each channel and also conduct advanced analyses of brain function, including power symmetry (the distribution of power between the left and right hemispheres) [34], coherence, (the level of synchronization between different brain regions), cordance (a measure of cerebral metabolism) [35,36,37,38], and source localization (the identification of the origin of specific EEG frequencies within the brain) [39,40,41].

Guided by the literature on the association between specific qEEG anomalies and psychopathology, the present narrative review synthesizes candidate findings that may help frame brain-behavior relationships across psychiatric conditions. Rather than implying transdiagnostic convergence or immediate clinical actionability, our aim was to highlight examples of recurring patterns and intervention-relevant hypotheses, while emphasizing that the evidence base remains heterogeneous and method-dependent [24,25,42,43,44].

## 2. Methods

A structured search of the literature was conducted on 31 January 2026 by employing the *Europe PMC* library to identify publications relevant to qEEG applications in psychiatry and neurology. We limited results to English-language records with an available abstract and included original human research (observational/comparative designs) and clinical trials with no age restrictions. To increase specificity for clinically relevant outcomes, we further restricted results to studies in which the diagnostic/biomarker intent was indicated in the title and/or abstract (e.g., terms related to biomarkers, diagnosis, prediction, indicators, or predictive value). Preprints and secondary literature (reviews, systematic reviews, and meta-analyses) were excluded. Finally, studies were screened to include both female and male participants.

By combining Artificial Intelligence (AI) technology (https://chatgpt.com) and manual adjustments the following search string was generated: ((Electroencephalography OR “quantitative electroencephalography” OR qEEG OR EEG OR electroencephalogr*) AND (psychiatry OR psychiatric OR “mental disorder*” OR psychopatholog*) AND (biomarker* OR diagnos* OR diagnosis OR predict* OR indicator* OR “predictive value”)) AND (TITLE:biomarker* OR TITLE:diagnos* OR TITLE:diagnosis OR TITLE:predict* OR TITLE:indicator* OR TITLE:”predictive value” OR ABSTRACT:biomarker* OR ABSTRACT:diagnos* OR ABSTRACT:diagnosis OR ABSTRACT:predict* OR ABSTRACT:indicator* OR ABSTRACT:”predictive value”) AND HAS_ABSTRACT:y AND LANG:eng AND KW:”Humans” AND (PUB_TYPE:”Clinical Trial” OR PUB_TYPE:”Randomized Controlled Trial” OR PUB_TYPE:”Controlled Clinical Trial” OR PUB_TYPE:”Observational Study” OR PUB_TYPE:”Comparative Study”) NOT SRC:PPR NOT (PUB_TYPE:”Review” OR PUB_TYPE:”Systematic Review” OR PUB_TYPE:”Meta-Analysis”) NOT (KW:”Animals” NOT KW:”Humans”) NOT (TITLE:mouse OR TITLE:mice OR TITLE:rat OR TITLE:murine OR TITLE:zebrafish OR ABSTRACT:mouse OR ABSTRACT:mice OR ABSTRACT:rat OR ABSTRACT:murine OR ABSTRACT:zebrafish).

## 3. Results

The search identified 1934 records, which were used to map the scope of the literature; however, given the narrative nature of the review, no formal systematic screening flow was undertaken. In the sections that follow, we present selected qEEG measures that may be informative as adjunctive markers, while emphasizing current limitations related to reproducibility, disorder specificity, normative standardization, and external validation. The reader should also bear in mind that the studies reported below are heterogeneous with respect to design, population characteristics, age range, sample size, recording context, preprocessing pipeline, and analytical methods. Accordingly, the findings discussed and summarized (Table 1) should be interpreted as varying in evidential strength, with some patterns appearing relatively replicated, others remaining provisional, and many being sensitive to methodological context. Finally, recent methodological work shows that real-world EEG assessments may depend critically on preprocessing rigor and artifact management [25,45,46,47,48,49]. Accordingly, any clinical interpretation of qEEG findings must be tempered by awareness of signal-quality limitations and pipeline-dependent variability.

### 3.1. Spectral Slowing and Redistribution of EEG Power

One of the most consistently replicated qEEG findings across psychiatric and cognitive disorders is spectral slowing, typically expressed as increased delta and theta power accompanied by reduced alpha and beta activity. For example, resting-state evidence indicates that this pattern may differentiate individuals with mild cognitive impairment and Alzheimer’s disease (AD) from healthy controls, correlating with performance in cognitive tasks and disease severity [50,51]. Similar EEG slowing patterns have also been found in affective and psychotic disorders [15,52], supporting the hypothesis that spectral power redistribution reflects altered thalamo-cortical gating and reduced neurotransmission efficiency rather than disorder-specific symptoms [51,53].

### 3.2. Peak Alpha Frequency

Peak alpha frequency (PAF) is defined as the frequency at which alpha-band power is maximal in the resting EEG, typically assessed while the eyes are closed. It is well established that PAF is an index of key aspects of cortical activity, such as information processing speed and large-scale network efficiency [54]. Reductions in PAF have been associated with cognitive slowing, increased disease burden, and poorer functional outcomes across psychiatric and neurological conditions [55,56,57]. Importantly, because PAF is intra-individually stable and sensitive to pathological changes, it can be used for patient stratification, prognostic assessment, and longitudinal monitoring [50,58]. In this context, the use of individually estimated PAF may allow clinicians to capture meaningful neurophysiological nuances not adequately represented by symptom-based diagnostic categories alone [59,60].

### 3.3. Frontal Alpha Asymmetry

Similar to spectral slowing, frontal alpha asymmetry (FAA) may exhibit clinical relevance when interpreted within context, rather than as a disorder-specific metric. Changes in asymmetry patterns may reflect emotional reactivity, motivational bias, and treatment response in mood disorders, even when DSM-based diagnoses overlap [61]. Therefore, the FAA can potentially contribute to refining diagnostic formulations and treatment selection in patients presenting with mixed clinical profiles, particularly when depressive and anxiety symptoms coexist [61,62].

### 3.4. Functional Connectivity and Network-Level Metrics

The qEEG method may also be employed to explore large-scale brain network organization by statistically comparing signals recorded from spatially distinct scalp regions. In most clinical qEEG platforms, a commonly available measure of functional connectivity is “coherence”, a frequency-specific metric that reflects the consistency of coupling between two EEG signals [63]. Many qEEG software packages also implement phase-based connectivity measures, such as the “phase-locking” value and related synchronization indices, to describe the alignment of oscillatory activity between regions [64,65]. In some systems, indices based on “phase-lag” and interhemispheric connectivity metrics also allow for long-range connectivity analyses and the evaluation of coupling between homologous left/right regions, which may be useful to unveil lateralized dysfunction or hemispheric imbalances [66,67]. Empirical studies support the clinical relevance of these measures. For example, Hill et al. [68] demonstrated that a phase-lag index reflected frequency-specific changes following electroconvulsive therapy in patients with major depressive disorder (MDD), and Chen et al. [69] reported that phase-locking differentiated children with attention deficit hyperactivity disorder (ADHD) from typically developing controls. In adolescents with MDD and suicidal ideation, Ozger et al. [70] identified frequency-specific changes in resting-state EEG coherence when compared with controls, demonstrating that coherence may capture clinically meaningful network differences. Finally, Galkin et al. [71] showed reduced EEG interhemispheric coherence across multiple frequency bands in patients with depressive disorders, supporting the hypothesis that interhemispheric connectivity metrics may reveal lateralized or asymmetric network changes relevant to psychiatric presentation in this clinical population. Collectively, these studies support that coherence-based, phase-locking, phase-lag, and interhemispheric connectivity measures may detect clinically meaningful neurophysiological anomalies and treatment-related changes in psychiatric populations, and that their use as adjunctive markers may complement symptom-based diagnostic classifications.

### 3.5. Source Localization

Source-localization methods such as Low-Resolution Electromagnetic Tomography (LORETA) extend conventional qEEG reach by computing an estimate of the cortical generators of the electrical activity recorded at the scalp level [72,73]. This is clinically relevant because source-derived markers can map heterogeneous symptom presentations onto network-level patterns [50,74]. For example, this approach has been shown to reliably distinguish between mild cognitive impairment (MCI) and AD-like profiles, or detect neurodegeneration changes throughout the AD continuum [50].

In patients with affective disorders, the LORETA method has been used to identify source generators of FAA, illustrating how source localization can refine surface qEEG data interpretation [74]. More broadly, recent methodological studies support LORETA-derived current density measures [73] and suggest that source analysis performed against normative templates may provide plausible activity patterns in routine clinical settings [75].

### 3.6. qEEG Markers for Treatment Monitoring

The highest clinical value of the qEEG method may lie in its ability to objectively measure neurophysiological changes. In patients with dementia, for example, studies have shown a link between pharmacotherapy and frequency-specific changes in the EEG [76,77], some of which may accompany symptomatic improvement following pharmacotherapy [76,78]. Comparable findings have been shown in MDD, where changes in EEG frequency-specific activity, connectivity, and complexity may accompany or predict clinical response to antidepressant treatment [79,80]. Notably, such EEG changes may precede overt symptomatic improvement [81], suggesting that closely monitoring qEEG metrics may contribute to minimizing adverse effects due to trial-and-error treatment strategies. Together, these findings underscore the potential role of the qEEG method in longitudinal monitoring, offering clinicians objective evidence to complement symptom ratings and clinical judgment.

## 4. Examples of qEEG Features Across Neuropsychiatric Disorders

### 4.1. Anxiety

Just as the DSM-5 considers anxiety as a multifactorial mood disorder driven by psychological, environmental, biological, and/or genetic factors [82], anxiety control can also be achieved via multifactorial forms, altogether exerting their modulatory influence on temporal beta activation [83]. Also, while alpha frontal asymmetries are often found in anxious individuals [84], diverging patterns can be observed [85]. In this context, some studies have failed to detect significant differences between anxious patients and controls, suggesting that alpha-based findings are inconsistent and may be strongly context-, sample-, and method-dependent [86,87]. In line with this, alpha band anomalies may not be detected in some patients with anxiety disorders, where instead abnormal elevations in gamma activity have been suggested to reflect pathological worry associated with intense negative emotions [88]. Importantly, while anxiety is often found in depressed patients, different EEG anomalies can be found in patients with anxiety disorder and patients with MDD [89], suggesting that different neurobiological correlates of apparently similar symptoms may require different interventions.

Promising support for the use of the qEEG method with anxiety patients is offered by machine learning studies. For example, recent research with individuals diagnosed with generalized anxiety disorder (GAD) found that patients exhibited a significant increase in beta (13–30 Hz) rhythm and a decrease in alpha1 (8–10 Hz) rhythm of power spectral density (PSD), together with reduced long-range functional connectivity between frontal and other brain areas in all frequency bands. These features contributed to reaching a classification performance with high accuracy, sensitivity, and specificity [90]. Similarly, recent research by Liu et al. [91] developed a machine learning framework to classify GAD with high accuracy by extracting functional connectivity features from frontal EEG channels and by employing machine learning algorithms. More recent research has identified high-accuracy EEG-based classifiers that were demonstrated to distinguish between anxious and non-anxious individuals, highlighting how selecting appropriate feature extraction methods and machine learning algorithms is key to developing reliable automated EEG-based systems for anxiety detection [92]. However, despite promising classification results, recent reviews of machine-learning approaches to anxiety detection emphasize that methodological heterogeneity and limited generalizability currently constrain confidence in the clinical robustness of these models [93,94].

### 4.2. Depression

When considering the broad symptom heterogeneity in depressed persons, well-designed studies based on the qEEG method have consistently revealed several anomalies. Decreased delta power, increased high-frequency (>12 Hz) power, reduced interhemispheric coherence, and altered frontal EEG alpha asymmetry have been demonstrated [95,96]. Of note, recent research has shown a reduction in alpha and theta activity immediately after infusion of midazolam or ketamine. However, while midazolam also increased delta (post-infusion), only ketamine increased gamma (immediately post- and 2 h post-infusion). Importantly, regional- and frequency-specific ketamine-induced EEG changes were related to and predictive of decreases in depressive symptoms and suicidal ideation [97]. On the other hand, another study shows that while changes in very high frequency oscillations were detected in patients treated with a single dose of ketamine, changes in glutamatergic neurotransmission were not associated with the alleviation of depressive symptoms [98]. This should be considered in light of the recent FDA approval of novel pharmacotherapeutic strategies to treat depression [99], which still does not adequately account for the substantial neurophysiological variability seen in this disease. Importantly, recent advances in translational medicine emphasize how mathematical modeling based on multimodal measures, including EEG-based metrics, can increase confidence in the diagnosis of depression and in predicting a positive response to antidepressant treatment [100]. Strikingly, similar results have been shown in research where machine learning algorithms were integrated into consumer-grade devices, remarking how EEG-based automation in depression screening is commercially accessible [101], although accessibility alone does not establish clinical validity, reproducibility, or readiness for routine use [102].

### 4.3. Post-Traumatic Stress Disorder (PTSD)

Patients diagnosed with PTSD show reduced relative power of alpha rhythms [103] and elevated relative power of beta rhythms [104]. Imbalances in the alpha band are associated with sensory disinhibition, hypervigilance, and chronic hyperarousal [105]. Increased beta power in people with PTSD is linked to a general state of hyperarousal [106], sleep disturbances [107], or cognitive flexibility deficits [108]. Interestingly, other research in veterans with PTSD found only beta and theta but not delta or alpha anomalies when this group was compared with healthy individuals [109]. On the other hand, normalization of pathologically reduced alpha rhythmicity (“alpha rebound effect”) within key regions of the default mode network has been shown to lead to a decrease in hyperarousal in PTSD patients [110,111], while optimization of the ratio between theta and beta power (TBR) was found to alleviate anxiety associated with alcoholism [112,113,114]. Notably, machine learning research suggests that functional connectivity-informed algorithms may increase prediction accuracy [115]. Of high clinical relevance is also the evidence suggesting that the use of portable EEG devices, combined with machine learning analysis, may provide valuable insights into symptom severity in this clinical population [116]. However, these findings should be interpreted cautiously, as machine-learning studies in PTSD remain highly heterogeneous in design, features, validation strategies, or sampled populations, and portability alone does not establish clinical robustness or routine-readiness [117].

### 4.4. Addiction

While addiction is a heterogeneous disorder, beta activity has been found to be generally elevated in patients with opioid and alcohol addiction and suppressed in patients with internet addiction. Also, an increase in theta power has been found in alcohol addiction (suggesting an element of lowered impulse control) [118,119]. On the other hand, a recent review [120] indicates that individuals with internet addiction and gaming disorder diagnosed following DSM-5 criteria exhibited distinct neurophysiological markers. Specifically, the participants with internet addiction showed increased gamma vs. decreased beta and delta power. In contrast, individuals with gaming disorder exhibited elevated delta and theta vs. reduced beta power. Coherence analysis also demonstrated mid-to-high frequency anomalies in gaming disorder. Machine learning research has shed further light on the diagnosis of addiction disorders by highlighting the role of differential delta and beta source connectivity patterns [121], also paving the way to the development of systems for automated detection in clinical practice [122].

### 4.5. ADHD

The use of qEEG as a diagnostic aid for ADHD continues to receive attention from both the scientific community and mental health professionals. Children with reduced attentional capacity exhibit qEEG abnormalities in up to 80% of cases [123]. The most represented imbalances appear in the frontal/polar regions, most likely because of the disruption of thalamocortical and/or septal-hippocampal pathways [124,125]. Studies employing qEEG distinguished between asymptomatic children, children with learning disorders, and children with attention disorders, also suggesting profiles that are more likely to improve following treatment with stimulant medication [123]. This might be in line with recent machine learning research suggesting complex qEEG patterns in ADHD [126,127].

Patients diagnosed with ADHD often show a link between increased theta activity and executive function deficits [128,129]. Also, age-dependent qEEG changes have been shown to allow for a distinction between ADHD subtypes [130,131,132]. However, these imbalances may reflect different symptoms across ADHD patients, given the heterogeneity of this diverse population. For example, increased frontal theta activity may be differentially linked to suboptimal executive functions in boys or hyperactivity in age-matched girls [133]. Recent large-scale EEG analyses in children aged between 5 and 18 years have highlighted the complexity of isolating modality-specific alpha-band rhythms, such as occipital alpha and sensorimotor mu (a type of alpha brainwave typically recorded over the sensorimotor cortex) [134]. These rhythms, although prominent and functionally relevant, show developmental changes in waveform shape that are not captured by traditional spectral methods [134].

### 4.6. Schizophrenia

It is generally accepted that symptomatology in schizophrenia is strongly associated with altered structural and/or functional communication between distant brain networks [135,136,137]. As connectivity between the brain networks that regulate resting state has widely been found to be altered, high variability and often contrasting evidence becomes problematic. For example, patients with schizophrenia consistently show greater low-frequency (delta and theta) and lower alpha power when compared with healthy controls [15]. Additionally, a greater TBR may be found in patients with schizophrenia, when compared to controls [15]. Interestingly, a lower TBR was found to be associated with mind wandering in individuals with high but not low schizotypal traits, suggesting a differential relationship between mind wandering and brain function [138]. On the other hand, negative symptoms in schizophrenia are associated with reduced alpha power and reduced alpha coherence between hemispheres and between right parietal and frontal regions [139].

While these findings confirm the high heterogeneity of the disorder, recent machine learning research has shown promising prediction accuracy [140], and a randomized clinical trial exploring the role of neurofeedback training as a complementary intervention in rehabilitating patients with schizophrenia found that normalization of several qEEG metrics correlated with an improvement of positive, negative, and general symptomatology [141]. However, such results should be interpreted cautiously, as EEG-based schizophrenia models remain highly sensitive to dataset characteristics, preprocessing choices, feature selection, and validation strategy, with generalizability to routine clinical settings still unproven [142].

### 4.7. Cognitive Impairment

Accumulating evidence indicates that qEEG may reveal functional imbalances in the brain before neuronal degeneration is detected by standard neuroimaging [143]. For example, in patients with MCI and patients with dementia, qEEG may detect “EEG slowing”, i.e., an elevation of delta and theta power often accompanied by the suppression of posterior alpha power, suggesting that suboptimal thalamo-cortical and large-scale network coordination may underpin cognitive decline and disease severity [51]. This could be in line with the research showing that brain connectivity anomalies could differentially impact amnestic MCI when compared with healthy controls [144], and may support etiological differences between subtypes of prodromal dementia [145,146].

Importantly, qEEG may reveal the selective effects of pharmacotherapy throughout treatment in patients with dementia. For example, in a randomized placebo-controlled trial with AD patients, changes in alpha-band functional connectivity were associated with the beneficial effects of glutaminyl cyclase inhibitor PQ912 [76]. Similarly, a recent placebo-controlled pilot trial reported that the administration of sigma-2 receptor ligand CT1812 reduced theta activity and improved alpha connectivity in AD patients, suggesting that qEEG may index short-timescale treatment effects in this population [77]. Finally, a double-blind crossover proof-of-principle study in patients with vascular cognitive impairment found that methylphenidate and galantamine produced measurable cognitive effects alongside EEG changes [147].

Taken together, these findings suggest that qEEG may offer clinically meaningful insights into the functional integrity of large-scale brain networks in patients with cognitive impairment. When qEEG changes are evaluated alongside subjective reports, neuropsychological assessments and established biomarkers, the qEEG method could enhance diagnostic confidence, monitor disease progression, and contribute to tracking the neurophysiological effects of pharmacotherapy.

## 5. Clinical Validity, Reliability, and Current Regulatory/Reimbursement Context

In clinical settings, the evidence linking symptoms to qEEG changes is still uneven [148,149]. Also, machine learning models and modern signal-processing approaches may increase classification accuracy in research settings, induce overfitting and still need to be validated across multiple sites and devices [150,151]. Therefore, their clinical deployment still requires more research and testing in real-world populations [148,149].

While some commercial qEEG devices are FDA-cleared, uneven regulatory and reimbursement policies continue to limit its implementation in clinical practice [152,153]. For instance, some policy statements remark that while qEEG can unveil functional imbalances in the brain [132], it should not be used as a standalone diagnostic method and instead be integrated in a battery comprising behavioral, neuropsychological, and medical evaluations to ensure clinical relevance [154,155].

## 6. Implementation Considerations for Clinics

Successful clinical implementation of the qEEG method requires training, certification, and clear operational protocols. In this context, the International qEEG Certification Board (IQCB) recommendations [156] outline that documented competency for qEEG specialists can be an important safeguard against data misinterpretation [148,156].

Moreover, it becomes increasingly imperative for clinics and providers to adopt highly secure data storage measures, in line with local and global privacy protection policies [157,158,159]. While the rise of consumer-grade qEEG devices improves patient access, it also complicates compliance, as data storage standards often differ across jurisdictions [160,161].

The limitations of lower-channel-count systems and platforms must also be acknowledged. Simplified versions of qEEG devices, EEG sensing, and data analysis still require systematic research and official approval, which clinicians should openly discuss with their patients [162,163].

## 7. Recommendations for qEEG-Guided Interventions

While standardized acquisition protocols and preprocessing may increase reliability of the qEEG method [26,164], qEEG-guided interventions require critical interpretation of the data acquired, also in combination with neurobehavioral and clinical data [26,165]. In other words, while out-of-the-box protocols based on the interpretation of qEEG data may promise to simplify the day-to-day clinical workload, they might also limit treatment precision and should be used only as a template [166]. Therefore, in clinical practice, the interpretation of qEEG data should stimulate clinical reasoning by helping identify neurophysiological patterns underlying behavioral symptoms, improving therapeutic targeting, and tracking the effects of interventions on brain function [31,167,168].

## 8. Discussion

### 8.1. Impact of qEEG on Psychiatric Care

The present paper offers a short narrative overview of the literature, suggesting that the qEEG method may identify key frequency-specific brain anomalies and targets for intervention in patients with psychiatric disorders. We suggest that this approach could increase confidence in day-to-day psychiatric assessments, offering clinicians the opportunity to rely more on objectively measured patterns of brain activity rather than qualitative assessments [169]. One major benefit of incorporating qEEG into psychiatric care is its ability to uncover distinct neurophysiological patterns [170]. In this context, the interpretation of qEEG findings based on DSM guidelines should be questioned and revisited, as psychiatry advances toward precision medicine.

### 8.2. Practical Applications of qEEG

The literature discussed in the present paper suggests that qEEG-based assessments may enhance diagnostic accuracy and treatment outcomes, especially in those patients whose clinical profile is unlikely to be adequately described by DSM guidelines. Furthermore, qEEG provides complementary insights into intervention efficacy by tracking measurable shifts in neural activity. This data may contribute to minimizing adverse effects from suboptimal treatments and reducing distress in vulnerable patient populations [171,172].

One promising advancement in this field is the use of machine learning algorithms that combine EEG and other physiological indicators to enhance the reliability and replicability of results, suggesting that multimodal assessments are key to interpreting the symptoms presented by the patient and detecting sub-symptomatic imbalances. Moreover, technological advances now allow providers to administer such assessments in just minutes, in some cases even remotely, employing user-friendly technology while a dedicated specialist monitors the patient’s progress [173]. However, clinical reliability may be tempered by device-related limitations such as artifact susceptibility, signal-quality variability, incomplete validation, as well as by broader methodological challenges including feasibility, reliability, ecological validity, supervision level, and environmental distractions during home-based testing [20,24,25,173,174,175,176,177,178].

### 8.3. Practical Challenges for Wider qEEG Adoption

While growing evidence supports the clinical utility of qEEG in psychiatry, practical challenges must still be addressed to enable its broader integration into routine clinical practice. For example, interpreting qEEG data requires a high level of expertise [156]. For this reason, clinicians should always seek collaboration with specialists with a strong background in neurophysiology and qEEG analysis to ensure a reliable interpretation of the data acquired. This can be key to leveraging the full potential of the qEEG method, particularly when it comes to utilizing neurophysiological findings to craft targeted interventions. Moreover, qEEG services can be costly, and reimbursement for qEEG procedures remains inconsistent, which can act as a challenge for the less affluent [179]. Finally, universally standardized protocols for data acquisition and analysis across different qEEG platforms still need to be established to minimize inter-site reliability. Addressing these infrastructural challenges is an essential step in realizing the full potential of qEEG in clinical practice.

## 9. Limitations and Caution

It is well established that medications impact EEG activity, and inter-individual variability further contributes to heterogeneity in the induced changes [180,181,182,183]. Moreover, the risk of over-interpretation should be considered, as excessive confidence in transient or context-dependent deviations may lead to disproportionate clinical decisions, particularly when such imbalances are not monitored longitudinally throughout treatment [184]. Finally, as is often the case in advanced diagnostics, clinicians are advised to remain cautious about over-promising results. The use of balanced language, acknowledgment of uncertainty, and transparent discussion of methodological limitations are essential to maintaining credibility and protecting patients from unnecessary concern [185,186,187].

## 10. Conclusions

While the DSM provides useful guidelines to carry out and interpret interview- and questionnaire-based evaluations in mental health patients, there is ample evidence that the current diagnostic frameworks are inadequate for reliably tailoring treatments based on measurable neurophysiological imbalances, especially in the case of intricate psychiatric cases. In this context, the qEEG method may offer the opportunity to establish relationships between neurophysiological imbalances and behavioral disturbances. We suggest that complementing the diagnostic process with qEEG-based assessments could contribute to alleviating the distress often caused by ineffective treatments, which naturally may result from the implementation of trial-and-error methods commonly used in DSM-guided psychiatric care.

Patients too frequently experience frustration, serious adverse effects, and even the worsening of symptoms when interventions are not well-tailored to their actual neurophysiological needs. By providing a data-driven approach, the qEEG method could minimize these setbacks and offer clinicians the opportunity to make more informed decisions, ultimately leading to better therapeutic outcomes. Moreover, integrating qEEG-based evaluations into clinical routine could significantly impact psychiatric care by providing a more objective rationale to regularly monitor the effects of interventions. This proactive approach could not only enhance treatment but also reduce the likelihood of relapse or even the worsening of symptoms by more accurately adjusting treatments. In the long term, such an approach could dramatically improve patients’ experience, particularly in the case of chronic or treatment-resistant conditions.

To fully realize the potential of the qEEG method, future research should further focus on validating qEEG biomarkers across diagnostic categories and symptom dimensions, also in combination with neuromodulation techniques and personalized pharmacotherapeutic interventions.

In summary, the qEEG method offers a clinically deployable approach to augment psychiatric diagnosis with objective, neurophysiological information. As the field of translational medicine continues to produce increasingly more sophisticated technologies to measure brain function in real time, psychiatry can shift toward a more precise, proactive, and effective paradigm of mental healthcare.

## Figures and Tables

**Table 1:** Synthesis of the EEG/qEEG findings across selected psychiatric and neurocognitive conditions, organized by clinical context, recurring electrophysiological patterns, suggested clinical use, and suggested neuropsychological implications. The table is intended to highlight recurring trends across heterogeneous studies rather than definitive disorder-specific biomarkers.

Clinical Context	qEEG/EEG Pattern(s)	Suggested Clinical Use	Suggested Implications
Anxiety	Increased beta activity (especially in temporal regions) possible frontal alpha asymmetry and, in some cases, elevated gamma activity. Reduced long-range frontal functional connectivity may also appear.	Differentiate anxiety from superficially similar presentations, including depression. Classification of generalized anxiety disorder may be enhanced if spectral and connectivity markers are combined.	This pattern suggests heightened cortical arousal, maladaptive threat monitoring, impaired frontal control of impulsivity, and worry-related overactivation rather than a single uniform anxiety signature.
Depression	Decreased delta power, increased fast-frequency activity above 12 Hz, reduced interhemispheric coherence, and altered frontal alpha asymmetry. Additional state-related findings include reductions in alpha and theta following ketamine or midazolam, with ketamine also linked to increased gamma and region-specific electrophysiological shifts.	Identify depression-related neurophysiological subtypes, improve diagnostic confidence, and help predict antidepressant response.	Depression reflects marked neurophysiological heterogeneity rather than one uniform brain pattern. Abnormalities, may index disrupted arousal regulation, altered hemispheric balance, impaired network integration, and treatment-sensitive neurobiological states.
PTSD	Reduced alpha power and elevated beta power, with some studies also reporting theta abnormalities.	Characterize hyperarousal-related physiology, estimate symptom severity, and support biomarker-informed classification. EEG may also help identify treatment-relevant targets, including alpha normalization and theta/beta ratio optimization.	Sensory disinhibition, chronic hypervigilance, sleep-related dysregulation, and persistent cortical hyperarousal. Clinically, qEEG may help define individualized PTSD subtypes and guide regulation-focused interventions more precisely.
Addiction	Subtype-specific EEG variation rather than one uniform pattern. Alcohol and opioid addiction are often linked to increased beta, with alcohol addiction also showing increased theta. In contrast, internet addiction may show increased gamma with reduced beta and delta, while gaming disorder may show elevated delta/theta and reduced beta, sometimes with coherence abnormalities.	Differentiate addiction subtypes, identify impulse-control and arousal-related neurophysiological markers. Source connectivity and coherence measures may be useful for refining diagnostic discrimination.	Including altered arousal regulation, impaired inhibitory control, reward-related dysregulation, and network-level connectivity disturbances. Clinically, qEEG is most useful as a stratification tool that helps distinguish subtype-specific mechanisms and informs more individualized treatment planning rather than implying a single addiction signature.
ADHD	Fronto-polar abnormalities, most often increased theta activity in networks linked to executive control. Findings may also vary by sex, age, and subtype, with developmental changes in alpha and sensorimotor rhythms adding complexity.	Adjunctive diagnostic aid to help distinguish ADHD from typical development and learning disorders, identify subtype-relevant patterns, and estimate which individuals may respond better to stimulant medication.	Altered frontally mediated regulation of attention, executive control, and behavioral inhibition, likely involving disrupted thalamocortical and related pathways.
Schizophrenia	Increased low-frequency activity (delta and theta), reduced alpha power, elevated theta/beta ratio, and abnormal coherence (especially reduced interhemispheric and frontoparietal alpha coherence).	Characterize network dysregulation, support biomarker-informed classification, and potentially track treatment-related change.	Impaired integration across distributed brain networks, with abnormalities in resting-state regulation, attentional control, and hemispheric coordination. Reduced alpha and coherence may be especially relevant to negative symptoms, while elevated slow-wave activity may reflect inefficient cortical processing.
Cognitive Impairment	Increased delta and theta power, together with reduced posterior alpha power. Additional findings may include connectivity abnormalities, especially in amnestic MCI and dementia subtypes.	Adjunctive method for case formulation, stratification, longitudinal monitoring, and treatment tracking.	Reduced functional integrity of large-scale brain networks, with slowing and alpha suppression reflecting progressive inefficiency in cortical communication and cognitive regulation.

Abbreviations: EEG, electroencephalography; qEEG, quantitative electroencephalography; PTSD, post-traumatic stress disorder; ADHD, attention-deficit/hyperactivity disorder; MCI, mild cognitive impairment.

## Data Availability

No new data were created or analyzed in this study. Data sharing is not applicable to this article.

## Abbreviations

The following abbreviations are used in this manuscript:

**A–C**	
ADHD	Attention-Deficit/Hyperactivity Disorder
DSM	Diagnostic and Statistical Manual of Mental Disorders
DSM-5	Diagnostic and Statistical Manual of Mental Disorders, Fifth Edition
EEG	Electroencephalography
**F–G**	
fMRI	Functional Magnetic Resonance Imaging
FDA	U.S. Food and Drug Administration
GD	Gaming Disorder
GAD	Generalized Anxiety Disorder
HC	Healthy Controls
**I–M**	
IA	Internet Addiction
MCI	Mild Cognitive Impairment
MDD	Major Depressive Disorder
MRI	Magnetic Resonance Imaging
**N–Q**	
PSD	Power Spectral Density
PTSD	Post-Traumatic Stress Disorder
qEEG	Quantitative Electroencephalography
**R–T**	
TBR	Theta/Beta Ratio

## References

[1] American Psychiatric Association (2013). Diagnostic and Statistical Manual of Mental Disorders: DSM-5™.

[2] Battle D.E. (2013). Diagnostic and Statistical Manual of Mental Disorders (DSM). Codas.

[3] Regier D.A., Kuhl E.A., Kupfer D.J. (2013). The DSM-5: Classification and criteria changes. World Psychiatry.

[4] Harrison J.E., Weber S., Jakob R., Chute C.G. (2021). ICD-11: An international classification of diseases for the twenty-first century. BMC Med. Inform. Decis. Mak..

[5] Scala J.J., Ganz A.B., Snyder M.P. (2023). Precision Medicine Approaches to Mental Health Care. Physiology.

[6] Kapadia M., Desai M., Parikh R. (2020). Fractures in the framework: Limitations of classification systems in psychiatry. Dialogues Clin. Neurosci..

[7] Zbozinek T.D., Rose R.D., Wolitzky-Taylor K.B., Sherbourne C., Sullivan G., Stein M.B., Roy-Byrne P.P., Craske M.G. (2012). Diagnostic overlap of generalized anxiety disorder and major depressive disorder in a primary care sample. Depress. Anxiety.

[8] Hopwood M. (2023). Anxiety Symptoms in Patients with Major Depressive Disorder: Commentary on Prevalence and Clinical Implications. Neurol. Ther..

[9] Subramanian M., McAuliffe T., Agrawal H. (2023). Assessing Variability in Reporting Severity of the Same Symptom (Fatigue) in the Context of Different Psychiatric Syndromes. Cureus.

[10] McLoughlin G., Makeig S., Tsuang M.T. (2014). In search of biomarkers in psychiatry: EEG-based measures of brain function. Am. J. Med. Genet. B Neuropsychiatr. Genet..

[11] Abi-Dargham A., Moeller S.J., Ali F., DeLorenzo C., Domschke K., Horga G., Jutla A., Kotov R., Paulus M.P., Rubio J.M. (2023). Candidate biomarkers in psychiatric disorders: State of the field. World Psychiatry.

[12] Abi-Dargham A., Horga G. (2016). The search for imaging biomarkers in psychiatric disorders. Nat. Med..

[13] van Beek E.J.R., Kuhl C., Anzai Y., Desmond P., Ehman R.L., Gong Q., Gold G., Gulani V., Hall-Craggs M., Leiner T. (2019). Value of MRI in medicine: More than just another test?. J. Magn. Reson. Imaging.

[14] Fenton G.W. (1984). The electroencephalogram in psychiatry: Clinical and research applications. Psychiatr. Dev..

[15] Newson J.J., Thiagarajan T.C. (2018). EEG Frequency Bands in Psychiatric Disorders: A Review of Resting State Studies. Front. Hum. Neurosci..

[16] Verma R.K., Kaur S., David S.R. (2012). An instant diagnosis for depression by blood test. J. Clin. Diagn. Res..

[17] Chu C., Wei H., Zhu W., Shen Y., Xu Q. (2017). Decreased Prostaglandin D2 Levels in Major Depressive Disorder Are Associated with Depression-Like Behaviors. Int. J. Neuropsychopharmacol..

[18] Prichep L.S. (2005). Use of normative databases and statistical methods in demonstrating clinical utility of QEEG: Importance and cautions. Clin. EEG Neurosci..

[19] Keizer A.W. (2021). Standardization and Personalized Medicine Using Quantitative EEG in Clinical Settings. Clin. EEG Neurosci..

[20] Niso G., Romero E., Moreau J.T., Araujo A., Krol L.R. (2023). Wireless EEG: A survey of systems and studies. NeuroImage.

[21] Lopez-Gordo M.A., Sanchez-Morillo D., Pelayo Valle F. (2014). Dry EEG electrodes. Sensors.

[22] Liu Q., Yang L., Zhang Z., Yang H., Zhang Y., Wu J. (2023). The Feature, Performance, and Prospect of Advanced Electrodes for Electroencephalogram. Biosensors.

[23] Adams E.J., Scott M.E., Amarante M., Ramirez C.A., Rowley S.J., Noble K.G., Troller-Renfree S.V. (2024). Fostering inclusion in EEG measures of pediatric brain activity. npj Sci. Learn..

[24] Ronca V., Di Flumeri G., Giorgi A., Vozzi A., Capotorto R., Germano D., Sciaraffa N., Borghini G., Babiloni F., Aricò P. (2024). o-CLEAN: A novel multi-stage algorithm for the ocular artifacts’ correction from EEG data in out-of-the-lab applications. J. Neural Eng..

[25] Vincenzo R., Marianna C., Rossella C., Gianluca D.F., Andrea G., Daniele G., Gianluca B., Fabio B., Pietro A. (2025). Beyond the lab: Real-world benchmarking of wearable EEGs for passive brain-computer interfaces. Brain Inform..

[26] Thatcher R.W. (2010). Validity and Reliability of Quantitative Electroencephalography. J. Neurother..

[27] Gudmundsson S., Runarsson T.P., Sigurdsson S., Eiriksdottir G., Johnsen K. (2007). Reliability of quantitative EEG features. Clin. Neurophysiol..

[28] Valencia A.L., Froese T. (2020). What binds us? Inter-brain neural synchronization and its implications for theories of human consciousness. Neurosci. Conscious..

[29] Rapp P.E., Keyser D.O., Albano A., Hernandez R., Gibson D.B., Zambon R.A., Hairston W.D., Hughes J.D., Krystal A., Nichols A.S. (2015). Traumatic brain injury detection using electrophysiological methods. Front. Hum. Neurosci..

[30] Koberda J.L. (2021). QEEG as a Useful Tool for the Evaluation of Early Cognitive Changes in Dementia and Traumatic Brain Injury. Clin. EEG Neurosci..

[31] Livint Popa L., Dragos H., Pantelemon C., Verisezan Rosu O., Strilciuc S. (2020). The Role of Quantitative EEG in the Diagnosis of Neuropsychiatric Disorders. J. Med. Life.

[32] Buzsáki G. (2006). Rhythms of the Brain.

[33] Britton J.W., Frey L.C., Hopp J.L., Korb P., Koubeissi M.Z., Lievens W.E., Pestana-Knight E.M., St. Louis E.K., St. Louis E.K., Frey L.C. (2016). Electroencephalography (EEG): An Introductory Text and Atlas of Normal and Abnormal Findings in Adults, Children, and Infants.

[34] Hughes J.R., John E.R. (1999). Conventional and quantitative electroencephalography in psychiatry. J. Neuropsychiatry Clin. Neurosci..

[35] Iznak A.F., Iznak E.V. (2022). EEG Predictors of Therapeutic Responses in Psychiatry. Neurosci. Behav. Physiol..

[36] Hunter A.M., Leuchter A.F., Morgan M.L., Cook I.A. (2006). Changes in brain function (quantitative EEG cordance) during placebo lead-in and treatment outcomes in clinical trials for major depression. Am. J. Psychiatry.

[37] Bares M., Brunovsky M., Kopecek M., Novak T., Stopkova P., Kozeny J., Sos P., Krajca V., Höschl C. (2008). Early reduction in prefrontal theta QEEG cordance value predicts response to venlafaxine treatment in patients with resistant depressive disorder. Eur. Psychiatry.

[38] Leuchter A.F., Cook I.A., Lufkin R.B., Dunkin J., Newton T.F., Cummings J.L., Mackey J.K., Walter D.O. (1994). Cordance: A new method for assessment of cerebral perfusion and metabolism using quantitative electroencephalography. Neuroimage.

[39] Korb A.S., Hunter A.M., Cook I.A., Leuchter A.F. (2009). Rostral anterior cingulate cortex theta current density and response to antidepressants and placebo in major depression. Clin. Neurophysiol..

[40] Akdeniz G. (2016). Electrical source localization by LORETA in patients with epilepsy: Confirmation by postoperative MRI. Ann. Indian Acad. Neurol..

[41] Asadzadeh S., Yousefi Rezaii T., Beheshti S., Delpak A., Meshgini S. (2020). A systematic review of EEG source localization techniques and their applications on diagnosis of brain abnormalities. J. Neurosci. Methods.

[42] Albrecht B., Brandeis D., Uebel H., Heinrich H., Mueller U.C., Hasselhorn M., Steinhausen H.C., Rothenberger A., Banaschewski T. (2008). Action monitoring in boys with attention-deficit/hyperactivity disorder, their nonaffected siblings, and normal control subjects: Evidence for an endophenotype. Biol. Psychiatry.

[43] Hall M.H., Smoller J.W. (2010). A new role for endophenotypes in the GWAS era: Functional characterization of risk variants. Harv. Rev. Psychiatry.

[44] Tang Y., Chorlian D.B., Rangaswamy M., Porjesz B., Bauer L., Kuperman S., O’Connor S., Rohrbaugh J., Schuckit M., Stimus A. (2007). Genetic influences on bipolar EEG power spectra. Int. J. Psychophysiol..

[45] Rico-Olarte C., Eskofier B.M., Lopez D.M. (2025). EEG-cleanse: An automated pipeline for cleaning electroencephalography recordings during full-body movement. MethodsX.

[46] Kessler R., Enge A., Skeide M.A. (2025). How EEG preprocessing shapes decoding performance. Commun. Biol..

[47] Böttcher A., Wendiggensen P., Mückschel M., Hoffmann S., Buss C., Kölch M., Körte I., Li S.C., Mall V., Marschik P. (2026). Standardizing EEG preprocessing for cross-site integration—the CLEAN pipeline. NeuroImage.

[48] Balam V.P. (2025). Automated EEG signal processing: A comprehensive investigation into preprocessing techniques and sub-band extraction for enhanced brain-computer interface applications. J. Neurosci. Methods.

[49] Arpaia P., Cacciapuoti M., Cataldo A., Criscuolo S., De Benedetto E., Masciullo A., Pesola M., Schiavoni R. (2025). Assessing the Role of EEG Biosignal Preprocessing to Enhance Multiscale Fuzzy Entropy in Alzheimer’s Disease Detection. Biosensors.

[50] Cecchetti G., Agosta F., Basaia S., Cividini C., Cursi M., Santangelo R., Caso F., Minicucci F., Magnani G., Filippi M. (2021). Resting-state electroencephalographic biomarkers of Alzheimer’s disease. Neuroimage Clin..

[51] Meghdadi A.H., Stevanović Karić M., McConnell M., Rupp G., Richard C., Hamilton J., Salat D., Berka C. (2021). Resting state EEG biomarkers of cognitive decline associated with Alzheimer’s disease and mild cognitive impairment. PLoS ONE.

[52] Ranlund S., Nottage J., Shaikh M., Dutt A., Constante M., Walshe M., Hall M.-H., Friston K., Murray R., Bramon E. (2014). Resting EEG in psychosis and at-risk populations — A possible endophenotype?. Schizophr. Res..

[53] Uhlhaas P.J., Singer W. (2010). Abnormal neural oscillations and synchrony in schizophrenia. Nat. Rev. Neurosci..

[54] Pascucci D., Menétrey M.Q., Passarotto E., Luo J., Paramento M., Rubega M. (2025). EEG brain waves and alpha rhythms: Past, current and future direction. Neurosci. Biobehav. Rev..

[55] Zhao Y., Shi H., Kong W., Wang X., Wei W., Zhan Z., Xue X. (2025). Peak alpha frequency as an objective biomarker for cognitive assessment in post-stroke cognitive impairment. Front. Aging Neurosci..

[56] Puttaert D., Wens V., Fery P., Rovai A., Trotta N., Coquelet N., De Breucker S., Sadeghi N., Coolen T., Goldman S. (2021). Decreased Alpha Peak Frequency Is Linked to Episodic Memory Impairment in Pathological Aging. Front. Aging Neurosci..

[57] Ramsay I.S., Lynn P.A., Schermitzler B., Sponheim S.R. (2021). Individual alpha peak frequency is slower in schizophrenia and related to deficits in visual perception and cognition. Sci. Rep..

[58] Grandy T.H., Werkle-Bergner M., Chicherio C., Lövdén M., Schmiedek F., Lindenberger U. (2013). Individual alpha peak frequency is related to latent factors of general cognitive abilities. Neuroimage.

[59] Voetterl H., van Wingen G., Michelini G., Griffiths K.R., Gordon E., DeBeus R., Korgaonkar M.S., Loo S.K., Palmer D., Breteler R. (2023). Brainmarker-I Differentially Predicts Remission to Various Attention-Deficit/Hyperactivity Disorder Treatments: A Discovery, Transfer, and Blinded Validation Study. Biol. Psychiatry Cogn. Neurosci. Neuroimaging.

[60] Voetterl H.T.S., Sack A.T., Olbrich S., Stuiver S., Rouwhorst R., Prentice A., Pizzagalli D.A., van der Vinne N., van Waarde J.A., Brunovsky M. (2023). Alpha peak frequency-based Brainmarker-I as a method to stratify to pharmacotherapy and brain stimulation treatments in depression. Nat. Ment. Health.

[61] van der Vinne N., Vollebregt M.A., van Putten M., Arns M. (2017). Frontal alpha asymmetry as a diagnostic marker in depression: Fact or fiction? A meta-analysis. Neuroimage Clin..

[62] Arns M., Etkin A., Hegerl U., Williams L.M., DeBattista C., Palmer D.M., Fitzgerald P.B., Harris A., deBeuss R., Gordon E. (2015). Frontal and rostral anterior cingulate (rACC) theta EEG in depression: Implications for treatment outcome?. Eur. Neuropsychopharmacol..

[63] Kaminski M., Blinowska K.J. (2022). From Coherence to Multivariate Causal Estimators of EEG Connectivity. Front. Physiol..

[64] Roascio M., Canessa A., Trò R., Mattioli P., Famà F., Giorgetti L., Girtler N., Orso B., Morbelli S., Nobili F. (2022). Phase and amplitude electroencephalography correlations change with disease progression in people with idiopathic rapid eye-movement sleep behavior disorder. Sleep.

[65] Ren Z., Zhao Y., Han X., Yue M., Wang B., Zhao Z., Wen B., Hong Y., Wang Q., Hong Y. (2023). An objective model for diagnosing comorbid cognitive impairment in patients with epilepsy based on the clinical-EEG functional connectivity features. Front. Neurosci..

[66] Zhang Y., Wang K., Wei Y., Guo X., Wen J., Luo Y. (2022). Minimal EEG channel selection for depression detection with connectivity features during sleep. Comput. Biol. Med..

[67] Chang Y., Wang X., Liao J., Chen S., Liu X., Liu S., Ming D. (2024). Temporal hyper-connectivity and frontal hypo-connectivity within gamma band in schizophrenia: A resting state EEG study. Schizophr. Res..

[68] Hill A.T., Hadas I., Zomorrodi R., Voineskos D., Farzan F., Fitzgerald P.B., Blumberger D.M., Daskalakis Z.J. (2020). Modulation of functional network properties in major depressive disorder following electroconvulsive therapy (ECT): A resting-state EEG analysis. Sci. Rep..

[69] Chen C., Yang H., Du Y., Zhai G., Xiong H., Yao D., Xu P., Gong J., Yin G., Li F. (2021). Altered Functional Connectivity in Children with ADHD Revealed by Scalp EEG: An ERP Study. Neural Plast..

[70] Ozger C., Chumachenko S., McVoy M., Croarkin P.E., Doruk Camsari D. (2023). Evidence for Altered Electroencephalography Coherence in Depressed Adolescents with Suicidal Ideation and Behaviors. J. Child Adolesc. Psychopharmacol..

[71] Galkin S.A., Levchuk L.A., Simutkin G.G., Ivanova S.A., Bokhan N.A. (2023). Coherence of the electroencephalogram and peripheral markers of nerve tissue damage in depressive disorders. Zhurnal Nevrol. Psikhiatrii Im. SS Korsakova.

[72] Eom T.H. (2023). Electroencephalography source localization. Clin. Exp. Pediatr..

[73] Völker J.M., Arguissain F.G., Andersen O.K., Biurrun Manresa J. (2021). Variability and effect sizes of intracranial current source density estimations during pain: Systematic review, experimental findings, and future perspectives. Hum. Brain Mapp..

[74] Smith E.E., Cavanagh J.F., Allen J.J.B. (2018). Intracranial source activity (eLORETA) related to scalp-level asymmetry scores and depression status. Psychophysiology.

[75] Gomez-Tapia C., Bozic B., Longo L. (2025). Evaluation of EEG pre-processing and source localization in ecological research. Front. Neuroimaging.

[76] Briels C.T., Stam C.J., Scheltens P., Bruins S., Lues I., Gouw A.A. (2020). In pursuit of a sensitive EEG functional connectivity outcome measure for clinical trials in Alzheimer’s disease. Clin. Neurophysiol..

[77] Vijverberg E., de Haan W., Scheijbeler E., Hamby M.E., Catalano S., Scheltens P., Grundman M., Caggiano A.O. (2024). A Pilot Electroencephalography Study of the Effect of CT1812 Treatment on Synaptic Activity in Patients with Mild to Moderate Alzheimer’s Disease. J. Prev. Alzheimers Dis..

[78] Baakman A.C., Gavan C., van Doeselaar L., de Kam M., Broekhuizen K., Bajenaru O., Camps L., Swart E.L., Kalisvaart K., Schoonenboom N. (2022). Acute response to cholinergic challenge predicts long-term response to galantamine treatment in patients with Alzheimer’s disease. Br. J. Clin. Pharmacol..

[79] Wu W., Zhang Y., Jiang J., Lucas M.V., Fonzo G.A., Rolle C.E., Cooper C., Chin-Fatt C., Krepel N., Cornelssen C.A. (2020). An electroencephalographic signature predicts antidepressant response in major depression. Nat. Biotechnol..

[80] Strafella R., Chen R., Rajji T.K., Blumberger D.M., Voineskos D. (2022). Resting and TMS-EEG markers of treatment response in major depressive disorder: A systematic review. Front. Hum. Neurosci..

[81] de la Salle S., Jaworska N., Blier P., Smith D., Knott V. (2020). Using prefrontal and midline right frontal EEG-derived theta cordance and depressive symptoms to predict the differential response or remission to antidepressant treatment in major depressive disorder. Psychiatry Res. Neuroimaging.

[82] American Psychiatric Association (2014). DSM-5: Manual Diagnóstico y Estadístico de los Trastornos Mentales.

[83] Ribas V.R., Ribas R.G., Nobrega J.A., da Nobrega M.V., Especie J.A.A., Calafange M.T., Calafange C.O.M., Martins H.A.L. (2018). Pattern of anxiety, insecurity, fear, panic and/or phobia observed by quantitative electroencephalography (QEEG). Dement. Neuropsychol..

[84] Thibodeau R., Jorgensen R.S., Kim S. (2006). Depression, anxiety, and resting frontal EEG asymmetry: A meta-analytic review. J. Abnorm. Psychol..

[85] Harpfer K., Spychalski D., Kathmann N., Riesel A. (2021). Diverging patterns of EEG alpha asymmetry in anxious apprehension and anxious arousal. Biol. Psychol..

[86] Harrewijn A., Van der Molen M.J.W., Westenberg P.M., Putman P. (2018). Frontal alpha asymmetry and anxiety: The role of behavioral inhibition and attentional control. J. Exp. Psychopathol..

[87] Harrewijn A., Van der Molen M.J.W., Westenberg P.M., Putman P. (2016). Putative EEG measures of social anxiety: Comparing frontal alpha asymmetry and delta-beta cross-frequency correlation. Cogn. Affect. Behav. Neurosci..

[88] Oathes D.J., Ray W.J., Yamasaki A.S., Borkovec T.D., Castonguay L.G., Newman M.G., Nitschke J. (2008). Worry, generalized anxiety disorder, and emotion: Evidence from the EEG gamma band. Biol. Psychol..

[89] Zhang Y., Lei L., Liu Z., Gao M., Liu Z., Sun N., Yang C., Zhang A., Wang Y., Zhang K. (2022). Theta oscillations: A rhythm difference comparison between major depressive disorder and anxiety disorder. Front. Psychiatry.

[90] Shen Z., Li G., Fang J., Zhong H., Wang J., Sun Y., Shen X. (2022). Aberrated Multidimensional EEG Characteristics in Patients with Generalized Anxiety Disorder: A Machine-Learning Based Analysis Framework. Sensors.

[91] Liu W., Zhou B., Li G., Luo X. (2024). Enhanced diagnostics for generalized anxiety disorder: Leveraging differential channel and functional connectivity features based on frontal EEG signals. Sci. Rep..

[92] Aldayel M., Al-Nafjan A. (2024). A comprehensive exploration of machine learning techniques for EEG-based anxiety detection. PeerJ Comput. Sci..

[93] Taskynbayeva M., Gutoreva A. (2025). Machine learning approaches to anxiety detection: Trends, model evaluation, and future directions. Front. Artif. Intell..

[94] Ostojic D., Lalousis P.A., Donohoe G., Morris D.W. (2024). The challenges of using machine learning models in psychiatric research and clinical practice. Eur. Neuropsychopharmacol..

[95] Diego M.A., Field T., Hernandez-Reif M. (2001). CES-D depression scores are correlated with frontal EEG alpha asymmetry. Depress. Anxiety.

[96] Liu X., Zhang H., Cui Y., Zhao T., Wang B., Xie X., Liang S., Sha S., Yan Y., Zhao X. (2024). EEG-based major depressive disorder recognition by neural oscillation and asymmetry. Front. Neurosci..

[97] de la Salle S., Phillips J.L., Blier P., Knott V. (2022). Electrophysiological correlates and predictors of the antidepressant response to repeated ketamine infusions in treatment-resistant depression. Prog. Neuropsychopharmacol. Biol. Psychiatry.

[98] Murphy N., Tamman A.J.F., Lijffijt M., Amarneh D., Iqbal S., Swann A., Averill L.A., O’Brien B., Mathew S.J. (2023). Neural complexity EEG biomarkers of rapid and post-rapid ketamine effects in late-life treatment-resistant depression: A randomized control trial. Neuropsychopharmacology.

[99] Ko K., Kopra E.I., Cleare A.J., Rucker J.J. (2023). Psychedelic therapy for depressive symptoms: A systematic review and meta-analysis. J. Affect. Disord..

[100] Watts D., Pulice R.F., Reilly J., Brunoni A.R., Kapczinski F., Passos I.C. (2022). Predicting treatment response using EEG in major depressive disorder: A machine-learning meta-analysis. Transl. Psychiatry.

[101] Suzuki K., Laohakangvalvit T., Sugaya M. (2024). Machine-Learning-Based Depression Detection Model from Electroencephalograph (EEG) Data Obtained by Consumer-Grade EEG Device. Brain Sci..

[102] Torous J., Linardon J., Goldberg S.B., Sun S., Bell I., Nicholas J., Hassan L., Hua Y., Milton A., Firth J. (2025). The evolving field of digital mental health: Current evidence and implementation issues for smartphone apps, generative artificial intelligence, and virtual reality. World Psychiatry.

[103] Clancy K., Ding M., Bernat E., Schmidt N.B., Li W. (2017). Restless ‘rest’: Intrinsic sensory hyperactivity and disinhibition in post-traumatic stress disorder. Brain.

[104] Huang M.X., Yurgil K.A., Robb A., Angeles A., Diwakar M., Risbrough V.B., Nichols S.L., McLay R., Theilmann R.J., Song T. (2014). Voxel-wise resting-state MEG source magnitude imaging study reveals neurocircuitry abnormality in active-duty service members and veterans with PTSD. Neuroimage Clin..

[105] Nicholson A.A., Ros T., Frewen P.A., Densmore M., Theberge J., Kluetsch R.C., Jetly R., Lanius R.A. (2016). Alpha oscillation neurofeedback modulates amygdala complex connectivity and arousal in posttraumatic stress disorder. Neuroimage Clin..

[106] Kay D.B., Buysse D.J. (2017). Hyperarousal and Beyond: New Insights to the Pathophysiology of Insomnia Disorder through Functional Neuroimaging Studies. Brain Sci..

[107] Colombo M.A., Ramautar J.R., Wei Y., Gomez-Herrero G., Stoffers D., Wassing R., Benjamins J.S., Tagliazucchi E., van der Werf Y.D., Cajochen C. (2016). Wake High-Density Electroencephalographic Spatiospectral Signatures of Insomnia. Sleep.

[108] Popescu M., Popescu E.A., DeGraba T.J., Hughes J.D. (2023). Cognitive flexibility in post-traumatic stress disorder: Sustained interference associated with altered modulation of cortical oscillatory activity during task-switching. Neuroimage Clin..

[109] Begic D., Hotujac L., Jokic-Begic N. (2001). Electroencephalographic comparison of veterans with combat-related post-traumatic stress disorder and healthy subjects. Int. J. Psychophysiol..

[110] Ros T., Frewen P., Theberge J., Michela A., Kluetsch R., Mueller A., Candrian G., Jetly R., Vuilleumier P., Lanius R.A. (2017). Neurofeedback Tunes Scale-Free Dynamics in Spontaneous Brain Activity. Cereb. Cortex.

[111] Nicholson A.A., Densmore M., Frewen P.A., Neufeld R.W.J., Theberge J., Jetly R., Lanius R.A., Ros T. (2023). Homeostatic normalization of alpha brain rhythms within the default-mode network and reduced symptoms in post-traumatic stress disorder following a randomized controlled trial of electroencephalogram neurofeedback. Brain Commun..

[112] Peniston E.G., Kulkosky P.J. (1989). Alpha-theta brainwave training and beta-endorphin levels in alcoholics. Alcohol. Clin. Exp. Res..

[113] Peniston E.G., Kulkosky P.J. (1990). Alcoholic personality and alpha-theta brainwave training. Med. Psychother. Int. J..

[114] Peniston E.G., Kulkosky P.J. (1991). Alpha-theta brainwave neurofeedback for Vietnam veterans with combat-related post-traumatic stress disorder. Med. Psychother..

[115] Li Q., Coulson Theodorsen M., Konvalinka I., Eskelund K., Karstoft K.I., Bo Andersen S., Andersen T.S. (2022). Resting-state EEG functional connectivity predicts post-traumatic stress disorder subtypes in veterans. J. Neural. Eng..

[116] Peddi A., Sendi M.S.E., Minton S.T., Langhinrichsen-Rohling R., Hinojosa C.A., West E., Ressler K.J., Calhoun V.D., van Rooij S.J.H. (2025). Towards predicting posttraumatic stress symptom severity using portable EEG-derived biomarkers. Sci. Rep..

[117] Wu Y., Mao K., Dennett L., Zhang Y., Chen J. (2023). Systematic review of machine learning in PTSD studies for automated diagnosis evaluation. npj Ment. Health Res..

[118] Balconi M., Finocchiaro R., Canavesio Y. (2014). Reward-system effect (BAS rating), left hemispheric “unbalance” (alpha band oscillations) and decisional impairments in drug addiction. Addict. Behav..

[119] Balconi M., Angioletti L., Crivelli D. (2024). Integrating EEG biomarkers in the neurocognitive screening of executive functions in substance and behavioral addiction. Front. Psychiatry.

[120] Burleigh T.L., Griffiths M.D., Sumich A., Wang G.Y., Kuss D.J. (2020). Gaming disorder and internet addiction: A systematic review of resting-state EEG studies. Addict. Behav..

[121] Lee J.Y., Song M.S., Yoo S.Y., Jang J.H., Lee D., Jung Y.C., Ahn W.Y., Choi J.S. (2024). Multimodal-based machine learning approach to classify features of internet gaming disorder and alcohol use disorder: A sensor-level and source-level resting-state electroencephalography activity and neuropsychological study. Compr. Psychiatry.

[122] Chhetri B., Goyal L.M., Mittal M. (2023). How machine learning is used to study addiction in digital healthcare: A systematic review. Int. J. Inf. Manag. Data Insights.

[123] Chabot R.J., di Michele F., Prichep L., John E.R. (2001). The clinical role of computerized EEG in the evaluation and treatment of learning and attention disorders in children and adolescents. J. Neuropsychiatry Clin. Neurosci..

[124] Hong S.B. (2023). Thalamocortical functional connectivity in youth with attention-deficit/hyperactivity disorder. J. Psychiatry Neurosci..

[125] García-Avilés Á., Albert-Gascó H., Arnal-Vicente I., Elhajj E., Sanjuan-Arias J., Sanchez-Perez A.M., Olucha-Bordonau F. (2015). Acute oral administration of low doses of methylphenidate targets calretinin neurons in the rat septal area. Front. Neuroanat..

[126] Mao Y., Qi X., He L., Wang S., Wang Z., Wang F. (2025). Advanced machine learning techniques reveal multidimensional EEG abnormalities in children with ADHD: A framework for automatic diagnosis. Front. Psychiatry.

[127] Kim J.W., Kim B.N., Kim J.I., Yang C.M., Kwon J. (2025). Electroencephalogram (EEG) Based Prediction of Attention Deficit Hyperactivity Disorder (ADHD) Using Machine Learning. Neuropsychiatr. Dis. Treat..

[128] Guo J., Luo X., Li B., Chang Q., Sun L., Song Y. (2020). Abnormal modulation of theta oscillations in children with attention-deficit/hyperactivity disorder. Neuroimage Clin..

[129] Hermens D.F., Soei E.X., Clarke S.D., Kohn M.R., Gordon E., Williams L.M. (2005). Resting EEG theta activity predicts cognitive performance in attention-deficit hyperactivity disorder. Pediatr. Neurol..

[130] Clarke A.R., Barry R.J., McCarthy R., Selikowitz M. (2001). Age and sex effects in the EEG: Differences in two subtypes of attention-deficit/hyperactivity disorder. Clin. Neurophysiol..

[131] Rodrak S., Wongsawat Y. EEG brain mapping and brain connectivity index for subtypes classification of attention deficit hyperactivity disorder children during the eye-opened period. Proceedings of the 2013 35th Annual International Conference of the IEEE Engineering in Medicine and Biology Society (EMBC).

[132] Board I.Q.C. Summary of IQCB Recommended Guidelines for Quantitative EEG (QEEG) Report Writing. https://qeegcertificationboard.org/wp-content/uploads/IQCB-Guidelines-for-Report-Writing-03-16-2025.pdf.

[133] Hermens D.F., Kohn M.R., Clarke S.D., Gordon E., Williams L.M. (2005). Sex differences in adolescent ADHD: Findings from concurrent EEG and EDA. Clin. Neurophysiol..

[134] Bender A., Voytek B., Schaworonkow N. (2025). Resting-state Alpha and Mu Rhythms Change Shape across Development But Lack Diagnostic Sensitivity for Attention-Deficit/Hyperactivity Disorder and Autism. J. Cogn. Neurosci..

[135] Friston K.J. (1999). Schizophrenia and the disconnection hypothesis. Acta Psychiatr. Scand..

[136] Andreasen N.C. (1997). Linking mind and brain in the study of mental illnesses: A project for a scientific psychopathology. Science.

[137] McGuire P.K., Frith C.D. (1996). Disordered functional connectivity in schizophrenia. Psychol. Med..

[138] Zhang Q., Jia L.X., Cui J.F., Ye J.Y., Liu J.L., Lai W.H., Shi H.S., Yang T.X., Wang Y., Chan R.C.K. (2024). Relationship between theta/beta ratio and mind wandering in schizotypy. PsyCh J..

[139] Merrin E.L., Floyd T.C. (1996). Negative symptoms and EEG alpha in schizophrenia: A replication. Schizophr. Res..

[140] Goshvarpour A. (2025). Enhancing schizophrenia diagnosis through EEG frequency waves and information-based neural connectivity feature fusion. Biomed. Signal Process. Control.

[141] Markiewicz R., Markiewicz-Gospodarek A., Dobrowolska B., Loza B. (2021). Improving Clinical, Cognitive, and Psychosocial Dysfunctions in Patients with Schizophrenia: A Neurofeedback Randomized Control Trial. Neural Plast..

[142] Rahul J., Sharma D., Sharma L.D., Nanda U., Sarkar A.K. (2024). A systematic review of EEG based automated schizophrenia classification through machine learning and deep learning. Front. Hum. Neurosci..

[143] Simfukwe C., An S.S.A., Youn Y.C. (2025). Time-Frequency Domain Analysis of Quantitative Electroencephalography as a Biomarker for Dementia. Diagnostics.

[144] Zawiślak-Fornagiel K., Ledwoń D., Bugdol M., Grażyńska A., Ślot M., Tabaka-Pradela J., Bieniek I., Siuda J. (2024). Quantitative EEG Spectral and Connectivity Analysis for Cognitive Decline in Amnestic Mild Cognitive Impairment. J. Alzheimers Dis..

[145] Schumacher J., Taylor J.P., Hamilton C.A., Firbank M., Cromarty R.A., Donaghy P.C., Roberts G., Allan L., Lloyd J., Durcan R. (2020). Quantitative EEG as a biomarker in mild cognitive impairment with Lewy bodies. Alzheimers Res. Ther..

[146] Novak K., Chase B.A., Narayanan J., Indic P., Markopoulou K. (2021). Quantitative Electroencephalography as a Biomarker for Cognitive Dysfunction in Parkinson’s Disease. Front. Aging Neurosci..

[147] Leijenaar J.F., Groeneveld G.J., Klaassen E.S., Leeuwis A.E., Scheltens P., Weinstein H.C., van Gerven J.M.A., Barkhof F., van der Flier W.M., Prins N.D. (2020). Methylphenidate and galantamine in patients with vascular cognitive impairment-the proof-of-principle study STREAM-VCI. Alzheimers Res. Ther..

[148] Simmatis L., Russo E.E., Geraci J., Harmsen I.E., Samuel N. (2023). Technical and clinical considerations for electroencephalography-based biomarkers for major depressive disorder. npj Ment. Health Res..

[149] Huidobro N., Meza-Andrade R., Mendez-Balbuena I., Trenado C., Tello Bello M., Tepichin Rodriguez E. (2025). Electroencephalographic Biomarkers for Neuropsychiatric Diseases: The State of the Art. Bioengineering.

[150] Xu L., Xu M., Ke Y., An X., Liu S., Ming D. (2020). Cross-Dataset Variability Problem in EEG Decoding With Deep Learning. Front. Hum. Neurosci..

[151] Saeidi M., Karwowski W., Farahani F.V., Fiok K., Taiar R., Hancock P.A., Al-Juaid A. (2021). Neural Decoding of EEG Signals with Machine Learning: A Systematic Review. Brain Sci..

[152] Nuwer M.R., Buchhalter J., Shepard K.M. (2016). Quantitative EEG in attention-deficit/hyperactivity disorder. Neurol. Clin. Pract..

[153] Medicare C.f., Services M. (2019). Special EEG Tests.

[154] Wood G., Willmes K., Koten J.W., Kober S.E. (2024). Fat tails and the need to disclose distribution parameters of qEEG databases. PLoS ONE.

[155] Rodríguez-Buritica D.F., Rojas-Céspedes L.A., Correa-Álvarez L. (2019). Technical and statistical milestones and standards for construction, validation and/or comparison of Quantitative Electroencephalogram (qEEG) normative databases. J. Neurol. Neurother..

[156] Collura T., Cantor D., Chartier D., Crago R., Hartzoge A., Hurd M., Kerson C., Lubar J., Nash J., Prichep L.S. (2025). International QEEG Certification Board Guideline Minimum Technical Requirements for Performing Clinical Quantitative Electroencephalography. Clin. EEG Neurosci..

[157] Association A.P. Telepsychiatry Toolkit. https://www.psychiatry.org/psychiatrists/practice/telepsychiatry/toolkit.

[158] (2018). General Data Protection Regulation.

[159] U.S. Food and Drug Administration (FDA) (2020). Policy for Device Software Functions and Mobile Medical Applications.

[160] Ienca M., Fins J.J., Jox R.J., Jotterand F., Voeneky S., Andorno R., Ball T., Castelluccia C., Chavarriaga R., Chneiweiss H. (2022). Towards a governance framework for brain data. Neuroethics.

[161] Meng L., Jiang X., Huang J., Xu Y., Li S. (2023). User Identity Protection in EEG-based Brain-Computer Interfaces. IEEE Trans. Neural Syst. Rehabil. Eng..

[162] Ratti E., Waninger S., Berka C., Ruffini G., Verma A. (2017). Comparison of Medical and Consumer Wireless EEG Systems for Use in Clinical Trials. Front. Hum. Neurosci..

[163] Krigolson O.E., Williams C.C., Norton A., Hassall C.D., Colino F.L. (2017). Choosing MUSE: Validation of a Low-Cost, Portable EEG System for ERP Research. Front. Neurosci..

[164] Coburn K.L., Lauterbach E.C., Boutros N.N., Black K.J., Arciniegas D.B., Coffey C.E. (2006). The value of quantitative electroencephalography in clinical psychiatry: A report by the Committee on Research of the American Neuropsychiatric Association. J. Neuropsychiatry Clin. Neurosci..

[165] Chumachenko S.Y., McVoy M. (2023). A Narrative Review and Discussion of Concepts and Ongoing Challenges of qEEG in Mood Disorders. J. Affect. Disord. Rep..

[166] Amico F., Danev S. (2022). Protocol Creation in Neurofeedback Training: Multimodal Assessments, Target Measures and Automation. J. Syst. Integr. Neurosci..

[167] Patil A.U., Lin C., Lee S.H., Huang H.W., Wu S.C., Madathil D., Huang C.M. (2023). Review of EEG-based Neurofeedback as a Therapeutic Intervention Across Multiple Disorders. Neurosci. Biobehav. Rev..

[168] Kopańska M., Rydzik Ł., Błajda J., Sarzyńska I., Jachymek K., Pałka T., Ambroży T., Szczygielski J. (2023). Quantitative Electroencephalography (qEEG) and Its Application in the Diagnosis and Therapy of Neuropsychiatric Disorders. Brain Sci..

[169] Chmielewski M., Clark L.A., Bagby R.M., Watson D. (2015). Method matters: Understanding diagnostic reliability in DSM-IV and DSM-5. J. Abnorm. Psychol..

[170] Olbrich S., van Dinteren R., Arns M. (2015). Personalized Medicine: Review and Perspectives of Promising Baseline EEG Biomarkers in Major Depressive Disorder and Attention Deficit Hyperactivity Disorder. Neuropsychobiology.

[171] Papakostas G.I., Petersen T., Pava J., Masson E., Worthington J.J., Alpert J.E., Fava M., Nierenberg A.A. (2003). Hopelessness and suicidal ideation in outpatients with treatment-resistant depression: Prevalence and impact on treatment outcome. J. Nerv. Ment. Dis..

[172] Olin B., Jayewardene A.K., Bunker M., Moreno F. (2012). Mortality and suicide risk in treatment-resistant depression: An observational study of the long-term impact of intervention. PLoS ONE.

[173] Emish M., Young S.D. (2024). Remote Wearable Neuroimaging Devices for Health Monitoring and Neurophenotyping: A Scoping Review. Biomimetics.

[174] Welhaf M., Wilks H., Aschenbrenner A.J., Katteri S., Morris J.C., Hassenstab J.J. (2025). Impacts of Environmental Distractions and Interruptions on Unsupervised Digital Cognitive Assessments in Older Adults: Cognitive Ecological Momentary Assessment Study. JMIR Mhealth Uhealth.

[175] Tailby C., Chapman J.E., Pugh R., Holth Skogan A., Helmstaedter C., Jackson G.D. (2025). Applications of teleneuropsychology to the screening and monitoring of epilepsy. Seizure.

[176] Polk S.E., Öhman F., Hassenstab J., König A., Papp K.V., Schöll M., Berron D. (2025). A scoping review of remote and unsupervised digital cognitive assessments in preclinical Alzheimer’s disease. npj Digit. Med..

[177] Caporusso E., Melillo A., Perrottelli A., Giuliani L., Marzocchi F.F., Pezzella P., Giordano G.M. (2025). Current limitations in technology-based cognitive assessment for severe mental illnesses: A focus on feasibility, reliability, and ecological validity. Front. Behav. Neurosci..

[178] Bayat M., Safaie J., Mollazadeh Beidokhti S., Wallois F. (2026). A comprehensive review of EEG electrode technologies: Advancements, applications, and future directions. Sens. Actuators A Phys..

[179] Hill C.E., Reynolds E.L., Burke J.F., Banerjee M., Kerber K.A., Magliocco B., Esper G.J., Skolarus L.E., Callaghan B.C. (2021). Increasing Out-of-Pocket Costs for Neurologic Care for Privately Insured Patients. Neurology.

[180] Hyun J., Baik M.J., Kang U.G. (2011). Effects of Psychotropic Drugs on Quantitative EEG among Patients with Schizophrenia-spectrum Disorders. Clin. Psychopharmacol. Neurosci..

[181] Blume W.T. (2006). Drug effects on EEG. J. Clin. Neurophysiol..

[182] Limotai C., Phayaph N., Pattanasilp B., Mokklaew J., Limotai N. (2020). Effects of antiepileptic drugs on electroencephalography (EEG). Epilepsy Behav..

[183] van Albada S.J., Rennie C.J., Robinson P.A. (2018). Variability of model-free and model-based quantitative measures of EEG. NeuroImage.

[184] Gelisse P., Benbadis S.R., Crespel A., Tatum W.O. (2024). Overcoming traps and pitfalls leading to misinterpretation of normal EEG variants and variation of the background activity. J. Neurol..

[185] Cox C., Fritz Z. (2025). In Defence of Causing Patients to Worry: Ethical Issues in the Communication of Diagnostic Uncertainty. Bioethics.

[186] Cox C., Hatfield T., Fritz Z. (2024). Role of communicating diagnostic uncertainty in the safety-netting process: Insights from a vignette study. BMJ Qual. Saf..

[187] Khazen M., Mirica M., Carlile N., Groisser A., Schiff G.D. (2023). Developing a Framework and Electronic Tool for Communicating Diagnostic Uncertainty in Primary Care. JAMA Netw. Open.

